# Bayesian approach for analysis of time-to-event data in plant biology

**DOI:** 10.1186/s13007-020-0554-1

**Published:** 2020-02-11

**Authors:** Jan F. Humplík, Jakub Dostál, Lydia Ugena, Lukáš Spíchal, Nuria De Diego, Ondřej Vencálek, Tomáš Fürst

**Affiliations:** 1 Department of Chemical Biology and Genetics, Centre of the Region Haná for Biotechnological and Agricultural Research, Faculty of Science, Palacký University, Šlechtitelů 27, 78371 Olomouc, Czech Republic; 2 Department of Mathematical Analysis and Application of Mathematics, Faculty of Science, Palacký University, 17. listopadu 12, 77900 Olomouc, Czech Republic

**Keywords:** Data analysis, Bayesian inference, Plant development, Statistics, Uncertainty, Time-to-event data, Survival analysis, Plant phenotyping

## Abstract

**Background:**

Plants, like all living organisms, metamorphose their bodies during their lifetime. All the developmental and growth events in a plant’s life are connected to specific points in time, be it seed germination, seedling emergence, the appearance of the first leaf, heading, flowering, fruit ripening, wilting, or death. The onset of automated phenotyping methods has brought an explosion of such time-to-event data. Unfortunately, it has not been matched by an explosion of adequate data analysis methods.

**Results and discussion:**

In this paper, we introduce the Bayesian approach towards time-to-event data in plant biology. As a model example, we use seedling emergence data of maize under control and stress conditions but the Bayesian approach is suitable for any time-to-event data (see the examples above). In the proposed framework, we are able to answer key questions regarding plant emergence such as these: (1) Do seedlings treated with compound A emerge earlier than the control seedlings? (2) What is the probability of compound A increasing seedling emergence by at least 5 percent?

**Conclusion:**

Proper data analysis is a fundamental task of general interest in life sciences. Here, we present a novel method for the analysis of time-to-event data which is applicable to many plant developmental parameters measured in field or in laboratory conditions. In contrast to recent and classical approaches, our Bayesian computational method properly handles uncertainty in time-to-event data and it is capable to reliably answer questions that are difficult to address by classical methods.

## Background

Reliable and robust tools for the analysis of high-throughput experimental data are reported to be the current bottleneck in plant phenotyping, breeding, and agricultural research [1]. Despite the current replication crisis and the dawning of “post p < 0.05 era”, this task is still not reflected enough in plant biology [2, 3]. There is overwhelming literature on how to analyze time-to-event data, mostly based on seed germination datasets. Ranal et al. [4] report that the number and diversity of mathematical expressions measuring various aspects of the germination process make any comparison difficult. They list a table of 19 parameters that have been used in the domain of germination assays only.

Most of the statistical treatment proceeds as follows [4, 5]: suppose we want to compare the median time to germination (*t*_*50*_) in two seed populations, *A* and *B*. We prepare five trays of seeds from population A (100 seeds per tray) and five trays of seeds from population B (100 seeds per tray). We collect the data and fit a parametric growth model (e.g., a logistic curve) to the data. Thus, we obtain five values for *t*_*50*_ for the seeds from population A and five values of *t*_*50*_ for the seeds from population B. Then we perform a *t-test* to compare whether the *t*_*50*_ value for the seeds from population A differs from the corresponding value for the seeds from population B. Notice that this procedure is *independent of the number of seeds in each tray!* The test has the same power, irrespective of whether each tray contains ten seeds or a million seeds. This is clearly inadequate, and yet this type of data evaluation is used more often than not [5].

One way out of this problem is to use the confidence intervals of the parameter estimates from the nonlinear regression [6]. It is clear that the width of the confidence intervals reflects the number of seeds in the tray. However, growth curve fitting by means of nonlinear regression is not appropriate for time-to-event data because the regression models assume a different underlying mechanism (see the Additional file 1 for a full explanation). Several authors noticed the inadequacy of nonlinear regression for growth models, most notably [7–10]. McNair et al. [7] correctly report that confidence intervals, a hypothesis test for parameter values, and a comparison of parameters for two or more groups that are provided by standard statistical software must be disregarded.

As has been reported previously, the correct approach is to embrace survival analysis methods for time-to-event data [7, 8, 10]. However, even survival analysis comes in two flavors: Classical (frequentist) and Bayesian. We offer a novel, general-purpose, easy-to-understand and flexible Bayesian tool to analyze any type of time-to-event data and to answer the most common scientific questions regarding this type of data.

Over the last century, survival analysis has provided us with many tools [11–13] to analyze time-to-event data (see also [7, 8] for a review). Most of them can readily be used in plant biology to analyze emergence or germination assays. In our case, the primary quantity of interest is the *emergence function F(t)*, which denotes the probability that a plant will emerge before time *t*. In practice, we often perform experiments to quantify the effect of the stressor (or a stimulant). To this end, a regression model must be used. The Cox proportional hazards (PH) model is the obvious candidate and the one that is used almost exclusively but it has a very strong assumption: it assumes that the effect of the predictor (e.g., a stressor) is *constant in time.* It is very likely that this assumption does not hold in many biological settings [14], especially in seedling emergence, which is connected with photomorphogenesis, one of the most dramatic periods in the life of plants. Moreover, we point out that *none of the classical methods* used for survival analysis is able to answer questions of type 2 above. To do that, we have to turn to Bayesian methods.

Lately, various machine learning (ML) methods have emerged in all fields that collect large amounts of data. It is natural to ask whether ML methods may be useful for answering the questions in the abstract. However, state-of-the-art ML methods deal with regression or classification (which are usually further used for prediction), but they have no tools to address the *uncertainty* of the result. Without the accompanying uncertainty, one cannot answer how probable it is that the classification offered by a ML tool is correct. From the point of view of statistics, ML method provide sophisticated but only point estimates. For most of the methods, estimates of variability are not yet available. Thus ML methods address a fundamentally different question than this article.

## Results

We illustrate the approach through a maize emergence experiment (see “Methods” for details). Let us first concentrate on a single tray of 110 seeds to introduce the Bayesian framework. The tray was observed every 2 h for 12 consecutive days. The number of plants that did not emerge at all is denoted by *M*. For each plant that emerged, we recorded during which 2-h interval it happened. Thus, for each interval *[t*_*i*_*, t*_*i*+*1*_*]*, we know the number *N*_*i*_ of plants that emerged during this interval. Here *t*_*0*_ = *0, t*_*2*_ = *2, t*_*3*_ = *4, …, t*_*144*_ = *288* because 288 h correspond to 12 days. Obviously

$$\sum\limits_{i = 0}^{T - 1} {N_{i} } = 110 - M,$$

where *T* = *144* denotes the number of 2-h intervals during the 12 days of the experiment. Let us denote the probability that a plant emerges before time *t* by *F(p, t)*, where by *p* we stress that the function depends on some parameters. If we fix the value of the parameters *p*, it is easy to write down the probability of the observed data. This probability (called the *likelihood function*) is a product of 110 terms because we assume that the emergence times of the individual plants are *independent.* For each plant that did not emerge at all, we multiply the likelihood by the term *[1 – F(p, t*_*T*_*)]*, which gives the probability that a plant will *not* emerge before the end of the experiment. If a plant emerged between *t*_*i*_ and *t*_*i*+*1*_, we multiply the likelihood by the term *[F(p, t*_*i*+*1*_*) – F(p, t*_*i*_*)]*, which gives the probability that a plant will emerge before *t*_*i*+*1*_ but not before *t*_*i*_. Altogether, the likelihood function can be written as

1 $$L(p) = [1 - F(p,t_{T} )]^{M} \prod\limits_{i = 0}^{T - 1} {[F(p,t_{i + 1} ) - F(p,t_{i} )]^{{N_{i} }} }$$

Note that the product runs over all the intervals, not all the plants that have emerged. Any interval with *N*_*i*_ = *0* will produce a unity and thus not alter the value of the product. Formula (1) is interpreted as the probability of the observed data, given the values of the parameters. Let us now change the point of view: consider the set of all possible values of the parameters. To each point in this parameter space, formula (1) assigns the probability of the observed data given these particular values of the parameters. Thus, formula (1) can be viewed as a function over the parameter space. Low values of *L* indicate values of parameters that are unlikely to produce the observed data. High values of *L* reveal the parameters which are likely to produce the observed data.

Here, a minor departure from standard parametric Bayesian models is needed. Let us assume that there are, in fact, two types of seedlings: those that would eventually emerge if we observed them for long enough, and those that would never emerge. Let us use *alpha* to denote the probability that a seedling will emerge eventually (i.e., *(1-alpha)* is the probability that it will never emerge). Then the probability that a seedling will emerge before time *t* can be written as

$$\begin{aligned} {\text{prob}}\left( {\text{seedling emerges before t}} \right) \, & = {\text{prob}}\left( {{\text{seedling emerges before t }}|{\text{ it is dead}}} \right){\text{ prob}}\left( {\text{it is dead}} \right) \, \\ & \quad + {\text{ prob}}\left( {{\text{seedling emerges before t }}|{\text{ it is alive}}} \right){\text{ prob}}\left( {\text{it is alive}} \right) \\ & = \, 0 \, * \, \left( {1 - {\text{ alpha}}} \right) \, + {\text{ F}}\left( {\text{t}} \right) \, *{\text{ alpha}} \\ \end{aligned}$$

Here *F(t)* again stands for a standard parametric growth model, e.g., the Gompertz distribution. The likelihood function (1) has to be altered accordingly: for each seedling which was not observed until the end of the experiment, we multiply the likelihood by the term

$$\left( {1 - alpha} \right) \, + \, alpha \, * \, \left( {1 \, {-} \, F\left( {p, \, t_{T} } \right)} \right)$$

This captures the probability that we did not see the plant emerge either because it was dead (the term *(1—alpha)*) or because it was alive but would have emerged after the end of the experiment (the term alpha * (1 – F(p, t_*T*_*))*). If a plant emerged between *t*_*i*_ and *t*_*i*+*1*_, we multiply the likelihood by the term

$$alpha* \, \left[ { \, F\left( {p, \, t_{i + 1} } \right) \, {-} \, F\left( {p, \, t_{i} } \right) \, } \right],$$

which gives the probability that the plant was alive and emerged before *t*_*i*+*1*_ but not before *t*_*i*_. Altogether, the likelihood function becomes

2 $$L(p) = [1 - \alpha F(p,t_{T} )]^{M} \prod\limits_{i = 0}^{T - 1} {[\alpha F(p,t_{i + 1} ) - \alpha F(p,t_{i} )]^{{N_{i} }} }$$

We will be ready to answer questions 1 and 2 once we understand how to treat the uncertainty of the parameter estimates provided by (2). Let us use the Gompertz model, which assumes

$$F\left( t \right) = \exp \left[ { - \exp \left( {B\left( {t - C} \right)} \right)} \right]$$

**Fig. 1:**
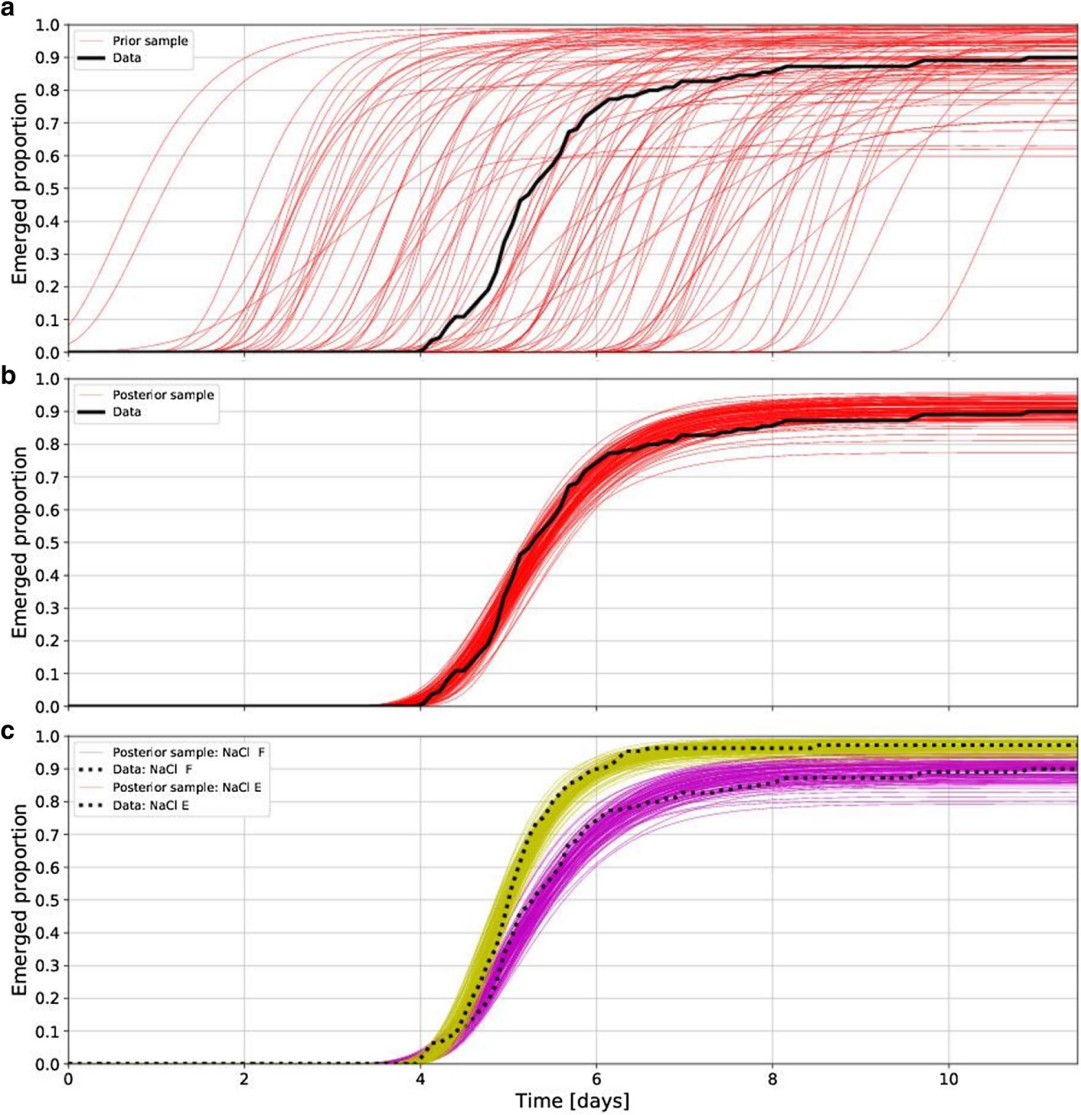
**a** Hundred samples of the possible emergence curves drawn from the prior distribution of the parameters. The data are shown in black but unused at this stage. Note that all the curves are increasing—this is a prior knowledge built into the model. **b** One hundred samples of the possible emergence curves drawn from the posterior. The difference between **a** and **b** shows how much our belief about the parameters of the emergence curve changed as a result of observing the data (shown in black). **c** Comparison of two emergence curves by means of sampling from the posterior. One hundred samples shown for each variant. See “Methods” for a description of the experimental design

There are three parameters to be estimated: the emergence yield *alpha*, emergence uniformity *B*, and emergence half-time *C*. Bayesian inference makes us spell out what we think about these parameters before we perform the experiment. Thus, each of the parameters is assigned a *prior distribution* which quantifies our degree of belief in the parameter’s values. Since *alpha* must lie in the interval *[0,1],* we assume its prior distribution to be the beta distribution, e.g., with the parameters [1, 9]. *B* and *C* are assumed to have a Gaussian prior with mu_B_ = 2, std_B_ = 0.5, and mu_C_ = 5, std_C_ = 2. Figure 1a shows a hundred samples of emergence curves drawn from this prior distribution.

After the data are observed, the prior distribution is updated according to Bayes’s Law, which states that

3 $${\text{Posterior}}\left( {\text{p}} \right) \, = {\text{ prior}}\left( {\text{p}} \right) \, *{\text{ likelihood}}\left( {{\text{p}},{\text{ data}}} \right) /{\text{ evidence}}\left( {{\text{data}}} \right).$$

The posterior represents the distribution of our degree of belief in the values of the parameters after observing the experimental data. The posterior directly quantifies the uncertainty of the values of the parameters together with the correlation among the parameters. Once the posterior is computed, all questions of the type 1 and 2 can easily be answered. The likelihood function in Eq. (3) has already been introduced and it is given by Eq. (2). The evidence in the denominator of (3) represents a normalization factor that is independent of the parameters and which need not be computed. Omitting the denominator, one computes an un-normalized posterior which is then rescaled to represent a probability density function (i.e., it is rescaled to a unit integral over the parameter space).

Let us now use Eq. (3) together with the likelihood function given by (2) to obtain the posterior. Figure 1b shows 100 emergence curves with parameters drawn from the posterior given by (3). We can observe that our vague initial belief in the shape of the emergence curve (shown in Fig. 1) was updated by the data and resulted in a fairly precise estimate of the emergence curve.

## Discussion

Let us now return to questions 1 and 2 from the introduction to illustrate practical application of Bayesian approach.

1. *Do seedlings treated with compound A emerge earlier than the control seedlings?*
  We have two sets of data, one for the control seeds and one for the primed seeds. We observe the emergence times and find the posterior distribution for the parameter *C* in both cases. Then we draw, e.g., a thousand samples of *C* from the posterior of the control *(C*^*1*^_*1*_* – C*^*1*^_*1000*_*)* and a thousand samples of *C* from the posterior of the treated seeds *(C*^*2*^_*1*_* – C*^*2*^_*1000*_*)*. The number of times *C*^*1*^ is less than *C*^*2*^ provides the estimate of the probability of seedlings from the primed seeds emerging earlier than seedlings from the control seeds. Figure 1c shows 100 emergence curves for both sets of data (data provided in the template file for the online evaluation web application described in the Additional file 1). The probability that *C*^*1*^ is less than *C*^*2*^ was estimated to be *p* = *0.01*, i.e., there is a more than 99% probability that seedlings from population A will emerge earlier than seedlings from the control population in the given conditions.
2. *What is the probability of compound E increasing seedling emergence in salinity (NaCl) by at least 5 percent with respect to compound F?*
  Here we need to estimate the probability that the parameter *alpha* is at least 5 percent higher in the seeds primed with E than in the seeds primed with F, sown both into NaCl containing substrate (see Fig. 1). We draw, e.g., a thousand samples *(alpha*^*1*^_*1*_* – alpha*^*1*^_*1000*_*)* from the posterior of the group F and a thousand samples *(alpha*^*2*^_*1*_* – alpha*^*2*^_*1000*_*)* from the posterior of the group E. We compute the number of times *alpha*^*2*^ ≥ 1.05*alpha^*1*^. This shows that there is a 73 percent probability that compound E increases seedling emergence by at least 5 percent with respect to compound F in the given conditions.

**Fig. 2:**
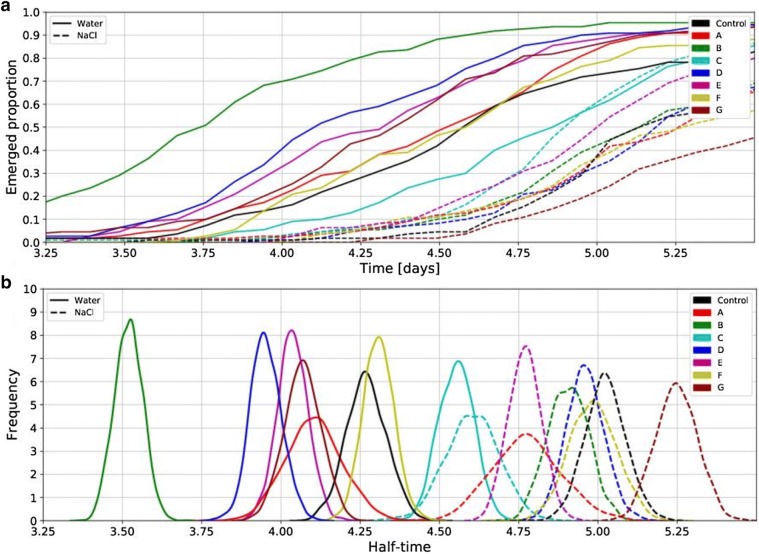
**a** Detail of empirical emergence curves for seeds primed with all the compounds that were tested. The significance of the differences among the curves are not obvious. **b** Posterior distributions for emergence half-time (parameter C in Table 1) across the entire population that was tested. The differences among the various compounds are clearly visible and can be quantified

It is difficult to imagine a relevant question which could not be answered in this straightforward way once the posterior is computed. The posterior distribution of the parameters can be used to compare a certain trait across the whole experiment. For example, Fig. 2b shows the plot of the posteriors for emergence half-time (parameter *C*) across the entire population that was tested. Sampling methods are needed to work with the posteriors but they are readily available nowadays in various software tools. We performed the sampling in Python [15, 16]. A free version of the simulation software is available at http://www.bayes4plants.com and described in the Additional file 1. The full code is freely available here.

**Table 1:** Summary of the mean value (expectation) of the posterior distribution of all three parameters for all the compounds that were tested

	Alpha	B	C
Control water	0.93	1.65	4.27
Control NaCl	0.96	1.54	5.03
A water	0.98	1.19	4.11
A NaCl	0.98	0.93	4.78
B water	0.97	2.33	3.52
B NaCl	0.97	1.63	4.91
C water	0.96	1.85	4.56
C NaCl	0.97	1.22	4.61
D water	0.99	2.01	3.95
D NaCl	0.96	1.70	4.96
E water	0.97	2.09	4.04
E NaCl	0.97	1.99	4.77
F water	0.97	2.11	4.31
F NaCl	0.91	1.40	4.99
G Water	0.98	1.81	4.07
G NaCl	0.93	1.58	5.25

The respective uncertainties are provided by the variance of the posterior distribution (see Fig. 2b for the uncertainty in parameter C)

## Conclusion

Proper data analysis is fundamental task of general interest in life sciences. Despite the current replication crisis and the dawning of “post p < 0.05 era” [2, 3], this task is still not reflected enough in plant biology. Here, we present a novel and reliable method for the analysis of time-to-event data which is applicable to many developmental parameters measured in field or indoor conditions, e.g. seed germination, seedling emergence, the appearance of the first leaf, heading, flowering, fruit ripening, wilting, or death. In contrast to recent and classical approaches, our Bayesian computational method properly handles uncertainty in time-to-event data and it is capable to reliably answer questions that are difficult to address by classical methods.

## Methods

Bayesian inference is general purpose method for handling uncertainty in experimental datasets. It should replace insufficient or biased “classical” statistical methods to provide more reliable data for better evidenced conclusions. Here we present an algorithm allowing such statistical analysis for time-to-event-data. The computational method is described above, whereas here we provide information about design of seedling emergence experiment that served as source of time-to-event data. However, this is only example of time-related dataset, Bayesian survival analysis can be performed on similar data-type coming from very different conditions (field or indoor) or different developmental stage of studied plant population. The data were obtained from automated screening of maize emergence. Seeds of the maize (*Zea mays* L.) hybrid Koblens (KWS Osiva s.r.o., Czech Republic) were sown into TEKU JP 3050/160T nursery trays. Each tray was watered to the full water capacity with tap water or a solution of 150 mM NaCl. Afterwards, all the trays were watered using 0.5 l of tap water every third day until the end of the experiment. The trays were randomly assigned to the control or the salt stress groups at the beginning of the experiment.

Together with the salt stress, a second effector was introduced into the experiment. The seeds were primed either with water (control) or one of seven potential stimulatory compounds (compounds A–G). The treatment was applied during the imbibition phase by soaking the seeds in the respective solution for 16 h before sowing. Each treatment was evaluated in control and in salt stress conditions. Thus 16 trays were evaluated, each containing 110 seeds. The trays were placed into a controlled condition chamber with a robotically driven arm holding an RGB camera [17]. Each tray was photographed every 2 h for 12 days. The images were stored in a database server and analyzed semi-automatically by an in-house image processing routine. For each maize seed, the time of its emergence (i.e., the moment of the appearance of the coleoptile above the soil) was recorded.

## Supplementary information

**Additional file 1.**1. Inadequacy of fitting of growth curves.; 2. Software

## Data Availability

The datasets during and/or analyzed during the current study available from the corresponding author on reasonable request. As we present here data analysis method, the sample data are free available to test in web http://www.bayes4plants.com. The code for the analysis is freely available here.
