# Supplementary material for: Bayesian approach for analysis of time-to-event data in plant biology
Source: Plant Methods. 2020 Feb 11;16:14. doi: 10.1186/s13007-020-0554-1 (PMC7011251; doi:10.1186/s13007-020-0554-1)
Supplement: Supplementary file 1 — Additional file 1.1. Inadequacy of fitting of growth curves.; 2. Software [file 13007_2020_554_MOESM1_ESM.docx]

**Additional material**

**1. Inadequacy of fitting of growth curves.**

Assume we collect the emergence data from a single homogeneous population of seeds and want to estimate the parameters of the emergence curve. At each point in time, the value of the empirical emergence curve is simply the number of seedlings that have already emerged. For illustration, we choose a simple logistic model, i.e., we assume that

S(t) = f(t) + noise (S1)

where S(t) denotes the observed number of plants that had emerged before time t, and f(t) denotes the theoretical model, i.e.,

f(t) = A / (1 + exp( –B(t - C))). (S2)

In this case, A captures the emergence yield, B the uniformity, and C the emergence half-time. A certain model for the noise is assumed, usually that the noise has the form of an *iid* (independent identically distributed) random variable. In this case, it is not difficult to fit the model to the data by means of nonlinear regression (which typically uses an iteratively re-weighted least squares method). This procedure provides an estimate of the parameters A, B, and C together with their confidence intervals. If need be, an estimate of the covariance matrix of the parameter estimates can be obtained.

However, simulated data reveal that the procedure described above is inadequate. Figure S1 shows simulated data (blue points) from model (S1), where the parameters in equation (S2) were taken to be A = 0.8, B = 2, and C = 3. The noise was drawn from a Gaussian distribution centered at zero with a variance of 0.003.


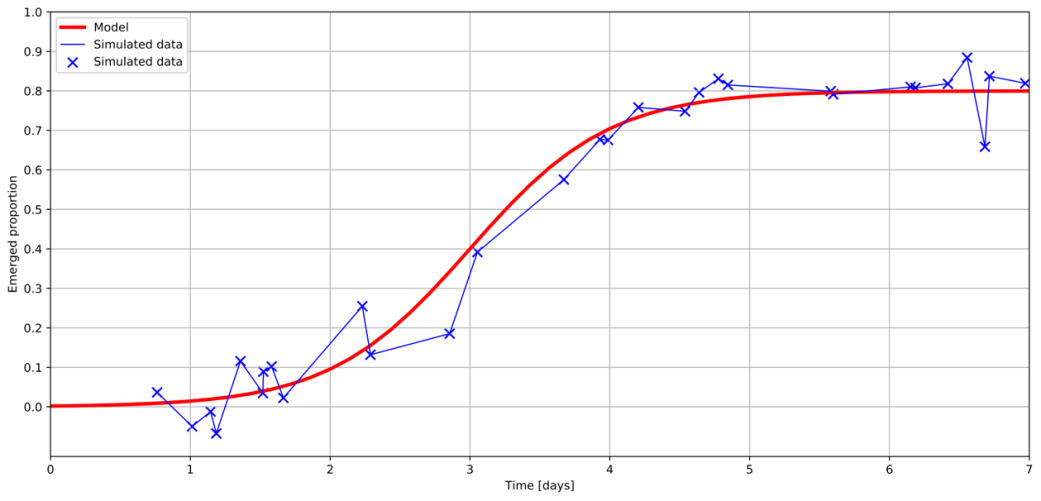


*Figure S1. Simulated data generated from model (S1). The model is inadequate in two ways: first, some data points may lie below zero (others may lie above 100%) and second, the data points are not monotonic in time.*

The simulated data in Figure S1 reveal two major drawbacks of model (S1). First, the assumption of additive *iid* noise in equation (S1) allows for data points below zero at the beginning and above 100% at the end of the curve. This is obviously absurd. Second, the simulated data are not monotonic in time. This suggests that some plants that have already emerged submerge again. The first failure of the model (data points below zero) could be repaired by assuming that the amplitude (variance) of the noise is proportional to the derivative of the model (i.e., small at both ends and large in the middle). However, this would make the model complicated and rather arbitrary. There is no obvious way to repair the second failure (the non-monotonicity of the data) within the scope of this approach. Despite the two obvious failures, this type of fitting time-to-events traits in plant biology is used very often [7]. This guarantees that the accompanying statistics are inadequate. Here we provide a paradigm-changing method that avoids these failures and offers a robust tool for the analysis of time-to-event data in plant biology.

**2. Software**

A free online tool using our algorithm is provided at the site [bayes4plants.com](http://www.bayes4plants.com) which enables evaluation of time-to-event data by means of the above described Bayesian methodology. The site provides a short explanatory text and sample data. The user may upload his/her own data provided they are in the same format as the sample data. The application allows the user to set the parameters of the prior distributions, visualize growth curves with parameters sampled from the prior and posterior distributions, and simulate the posterior distributions of the parameters of the parametric mixture model described above.

Numerical sampling of the posterior distributions is performed by means of the No-U-Turn Sampler (NUTS, an adaptive HMC sampler) which is initialized with the MAP estimate [15]. The simulation runs in Python3 using the PyMC3 package [16]. The package allows for the implementation of a custom likelihood (Equation 2) and supports the NUTS sampler.

The code is freely available [here](https://gist.github.com/DostalJ/73d985576fdfac926b9053b29f1e772e). The core section of the code is the definition of the model and its sampling. The relevant part of the code is reprinted here:

**class** **SurvivalLikelihood**(Continuous):

**def** __init__(self, alpha, k, tm, *args, **kwargs):

*"""*

*Custom time-to-event data likelihood.*

*"""*

super(SurvivalLikelihood, self).__init__(*args, **kwargs)

*# Three parameters of our model.*

self.alpha = alpha

self.k = k

self.tm = tm

**def** logp(self, pit_times, dt, end_time):

*"""*

*Obligatory definition of logarithm of the*

*probability used by pymc3 sampler.*

*"""*

n_pits = pit_times.shape[0]

events = pit_times[np.where((1 - tt.isnan(pit_times)).eval())]

n_events = events.shape[0]

gomp_t = gompertz(t=events, k=self.k, tm=self.tm)

gomp_t_dt = gompertz(t=events-dt, k=self.k, tm=self.tm)

g_diff = self.alpha*(gomp_t - gomp_t_dt)

lik1 = tt.sum(tt.log(g_diff))

lik2 = (n_pits - n_events) * tt.log(1 - self.alpha*gompertz(t=end_time, k=self.k, tm=self.tm))

**return** lik1 + lik2

**with** pm.Model() **as** model:

*# Prior definition*

alpha = pm.Beta('alpha', alpha=9, beta=1)

k = pm.Normal('k', mu=2, sd=0.5)

tm = pm.Normal('tm', mu=5, sd=2)

*# Likelihood definition*

likelihood = SurvivalLikelihood('likelihood', alpha=alpha, k=k, tm=tm,

observed={'pit_times': pit_times, 'dt': dt, 'end_time': end_time})

trace = pm.sample(1000, tune=500)
